# Comparator Data Characteristics and Testing Procedures for the Clinical Performance Evaluation of Continuous Glucose Monitoring Systems

**DOI:** 10.1089/dia.2023.0465

**Published:** 2024-03-28

**Authors:** Manuel Eichenlaub, Stefan Pleus, Martina Rothenbühler, Timothy S. Bailey, Lia Bally, Ronald Brazg, Daniela Bruttomesso, Peter Diem, Elisabet Eriksson Boija, Marion Fokkert, Cornelia Haug, Rolf Hinzmann, Johan Jendle, David C. Klonoff, Julia K. Mader, Konstantinos Makris, Othmar Moser, James H. Nichols, Kirsten Nørgaard, John Pemberton, Elizabeth Selvin, Loukia Spanou, Andreas Thomas, Nam K. Tran, Lilian Witthauer, Robbert J. Slingerland, Guido Freckmann

**Affiliations:** ^1^ Institut für Diabetes-Technologie, Forschungs- und Entwicklungsgesellschaft mbH an der Universität Ulm, Ulm, Germany.; ^2^ IFCC Scientific Division, Working Group on Continuous Glucose Monitoring.; ^3^ Diabetes Center Berne, Bern, Switzerland.; ^4^ AMCR Institute, Escondido, California, USA.; ^5^ Department of Diabetes, Endocrinology, Nutritional Medicine and Metabolism, Inselspital Bern, Bern University Hospital and University of Bern, Bern, Switzerland.; ^6^ Rainier Clinical Research Center, Renton, Washington, USA.; ^7^ Division of Metabolic Disease, Department of Medicine, University of Padua, Padua, Italy.; ^8^ Endokrinologie Diabetologie Bern, Bern, Switzerland.; ^9^ Equalis AB, Uppsala, Sweden.; ^10^ Department of Clinical Chemistry, Isala Clinics, Zwolle, The Netherlands.; ^11^ Roche Diabetes Care GmbH, Mannheim, Germany.; ^12^ School of Medical Sciences, Faculty of Medicine and Health, Örebro University, Örebro, Sweden.; ^13^ Diabetes Research Institute of Mills-Peninsula Medical Center, San Mateo, California, USA.; ^14^ Division of Endocrinology and Diabetology, Medical University of Graz, Graz, Austria.; ^15^ Clinical Biochemistry Department, KAT General Hospital, Athens, Greece.; ^16^ Department of Exercise Physiology and Metabolism, University of Bayreuth, Bayreuth, Germany.; ^17^ Department of Pathology, Microbiology and Immunology, Vanderbilt University Medical Center, Nashville, Tennessee, USA.; ^18^ Steno Diabetes Center Copenhagen, Herlev, Denmark.; ^19^ Department of Clinical Medicine, University of Copenhagen, Copenhagen, Denmark.; ^20^ Birmingham Women's and Children's Foundation Trust, Birmingham, United Kingdom.; ^21^ Department of Cardiovascular and Clinical Epidemiology, Johns Hopkins Bloomberg School of Public Health, Baltimore, Maryland, USA.; ^22^ Department of Endocrinology, Diabetes and Metabolism, Hellenic Red Cross Hospital, Athens, Greece.; ^23^ Independent Scientific Consulting, Pirna, Germany.; ^24^ Department of Pathology and Laboratory Medicine, University of California Davis Health, Sacramento, California, USA.

**Keywords:** Continuous glucose monitoring, Clinical performance evaluation, Standardization, Comparator data characteristics, Testing procedures, Glucose rate of change

## Abstract

Comparing the performance of different continuous glucose monitoring (CGM) systems is challenging due to the lack of comprehensive guidelines for clinical study design. In particular, the absence of concise requirements for the distribution of comparator (reference) blood glucose (BG) concentrations and their rate of change (RoC) that are used to evaluate CGM performance, impairs comparability. For this article, several experts in the field of CGM performance testing have collaborated to propose characteristics of the distribution of comparator measurements that should be collected during CGM performance testing. Specifically, it is proposed that at least 7.5% of comparator BG concentrations are <70 mg/dL (3.9 mmol/L) and >300 mg/dL (16.7 mmol/L), respectively, and that at least 7.5% of BG-RoC combinations indicate fast BG changes with impending hypo- or hyperglycemia, respectively. These proposed characteristics of the comparator data can facilitate the harmonization of testing conditions across different studies and CGM systems and ensure that the most relevant scenarios representing real-life situations are established during performance testing. In addition, a study protocol and testing procedure for the manipulation of glucose levels are suggested that enable the collection of comparator data with these characteristics. This work is an important step toward establishing a future standard for the performance evaluation of CGM systems.

## Introduction

In the recent past, the performance, in particular the accuracy, of continuous glucose monitoring (CGM) systems has improved sufficiently to enable nonadjunctive use for clinical decision-making.^1^ Together with the introduction of factory-calibration, that is, the redundancy of manual finger-stick calibration, in most systems, CGM is now an integral part of routine clinical diabetes management. Furthermore, targets for CGM-derived therapy metrics such as the “time in range” have been incorporated into national and international diabetes management guidelines,^2,3^ and have been used to determine the effectiveness of new diabetes therapies.^4^ Therefore, a rigorous and standardized evaluation of CGM system performance is crucial to ensure the safety and efficacy of CGM use in people with diabetes and the validity of CGM-based outcomes in clinical trials.

This article reviews the relevant literature and presents our expert proposal for characteristics of comparator data and the testing procedures during the clinical performance evaluation of CGM systems.

### Problem statement

Despite the widespread adoption of CGM system use, there are no comprehensive guidelines, regulatory or otherwise, for the study design of clinical CGM performance evaluations.^5^ Some, but not all, crucial elements of study design are covered in the POCT05 guideline put forward by the Clinical and Laboratory Standards Institute,^6,7^ and the requirements for “integrated” CGM (iCGM) systems set forth by the U.S. Food and Drug Administration (FDA).^8^ This has led to a wide range of study designs that can account for the discrepancies observed in the performance levels of the same CGM system.^9,10^ As a result, it is challenging to obtain a clear picture of the performance of any individual system and make meaningful comparisons between CGM systems.^10,11^

A crucial element of the study design is the testing procedures, especially for the deliberate manipulation of glucose levels. These procedures can create low and high blood glucose (BG) concentrations as well as fast and slow BG concentration changes during comparator (reference) data collection. These data are subsequently used to determine CGM system performance. This performance encompasses aspects such as analytical and clinical accuracy as well as the changes in accuracy over the sensor lifetime (stability) and the reliability of CGM system alerts.^10^ Therefore, the testing procedures need to be designed to enable the comprehensive assessment of these different aspects of performance. Furthermore, it has been shown that the characteristics of the comparator BG measurements, and therefore also the testing procedures for the manipulation of glucose levels, have a major influence on the observed performance of a CGM system. In particular, a potential deterioration of accuracy during hypoglycemia or periods of fast BG concentration changes has been observed.^12–16^ A testing procedure in which these conditions are underrepresented could lead to biased performance results, highlighting the need for standardized performance testing, especially with regard to the characteristics of the comparator data.

The aim of this article is to propose specific characteristics of the comparator measurements collected during CGM performance studies that include both the BG concentrations and their rate of change (RoC). These characteristics were selected based on an analysis of real-world CGM data and can ensure that all relevant glycemic situations are covered during performance testing. In addition, we suggest a study protocol and testing procedure capable of producing comparator data with the proposed characteristics, thus enabling a thorough investigation of CGM system performance. This article is a proposal from the Working Group on Continuous Glucose Monitoring established by the Scientific Division of the International Federation of Clinical Chemistry and Laboratory Medicine (IFCC)^11,17^ and other experts in the field of CGM performance testing.

### Literature background

Study protocols of CGM performance studies typically include the collection of comparator data in both in-clinic and free-living settings. In the free-living setting, subjects are instructed to measure their capillary BG levels multiple times per day with a home-use blood glucose monitoring (BGM) system. The advantage of this setting is that it represents CGM use under real-life conditions and that it can produce comparator data on every day of the sensor lifetime, which is important to assess the continued accuracy of the CGM system from insertion to removal. The disadvantage is that the distribution of comparator data is difficult to control and that the frequent measurements required for the determination of BG level RoCs are difficult to attain. This means that a robust and comprehensive estimation of accuracy and alert reliability from free-living data alone is not feasible. Furthermore, the measurement accuracy of home-use BGM systems is often inferior to laboratory-grade glucose analyzers. For these reasons, studies are designed to also include in-clinic sessions, where participants may spend several hours to multiple days at the investigational sites. Here, comparator measurements can be carried out every 5–15 min, typically for up to 12 h. Moreover, BG level dynamics can be manipulated through adaptation of insulin dosing/timing and consumption of specific meals.^10^

Our recent review of 129 clinical CGM performance studies published between 2002 and 2022 found that 73% of studies including in-clinic sessions reported a deliberate manipulation of glucose levels, although the glucose manipulation procedures were often described with insufficient detail.^10^ From the limited available information, there appear to be two main testing procedures targeting different glucose profiles during the in-clinic sessions.

First, there are studies that aim to produce either hypo- or hyperglycemic BG concentrations within a single session lasting between 8 and 12 h in total, but the actual time spent in hypo- or hyperglycemia is not reported.^18–22^ This approach appears to be recommended in the POCT05 guideline,^7^ although it likely limits RoCs because of a lack of change between low and high BG concentrations. Second, there exist studies that aim to induce both hypo- and hyperglycemic BG concentrations within a single in-clinic session, with some studies opting to start with hyperglycemia followed by hypoglycemia,^13,14,23–32^ while other studies use the reverse order,^33–35^ or choose the order based on the BG concentration at the start of the session.^15^ Inducing hypo- and hyperglycemic BG concentrations within the same session typically leads to higher RoCs.

The goal of the glucose manipulation, and thus the target characteristics of the comparator data distribution, is often not defined. Only a few articles state the vague rationale to produce comparator data covering a wide range of BG levels.^18–20,33,36–38^ In this context, the POCT05 guideline is more specific, recommending 8% of comparator values to be <80 mg/dL (4.4 mmol/L) and 5% to be >300 mg/dL (16.7 mmol/L).^7^ This recommendation was not found in the first edition of the guideline published in 2008,^6^ but was added to the second edition of the POCT05 guideline published in 2020.^7^ Its adoption in current studies is thus, so far, difficult to assess. However, a recent review, found that only 50% of the studies utilized for Conformité Européenne marking of CGM systems reported data on adults that satisfied both POCT05 distribution criteria.^5^ In contrast, the FDA iCGM requirements are more vague, only stating that “[…] clinical data must be obtained […] throughout the measuring range of the device.”^8^

The importance of producing rapidly changing BG concentrations is not mentioned in the current guidelines. Interestingly, the first edition of the POCT05 guideline recommended that “a sufficient number of points should be obtained at the extreme rates of change (< −1.5 mg/dL/min and >1.5 mg/dL/min),”^6^ without providing a precise percentage. This statement has been removed in the second edition.^7^ The majority of published articles also make no statement about the targeted BG concentration changes, with only some reports published more than 10 years ago explicitly mentioning the goal to assess CGM performance during rapidly falling and rising BG concentrations.^21,22,39^

## General **S**tudy **D**esign

### Study population

The elements of study design proposed in this article concern the performance evaluation of CGM systems in adults, intended to be used for therapeutic decision-making and integration into systems for automated insulin delivery (AID). The study population should thus reflect the intended use population of the CGM systems, that is, mainly people with type 1 diabetes, but also people with type 2 diabetes. To facilitate the collection of comparator data with the characteristics proposed in this article, we suggest that at least 75% of participants have type 1 diabetes. This suggestion agrees with the results of a recent literature review, where we found that on average 73.5% of participants had type 1 diabetes in studies including participants with both diabetes types.^10^

For CGM systems with other intentions of use, especially other intended use populations, this proposal may not be suitable, and different approaches may be needed.

### Study protocol

**FIG. 1.**
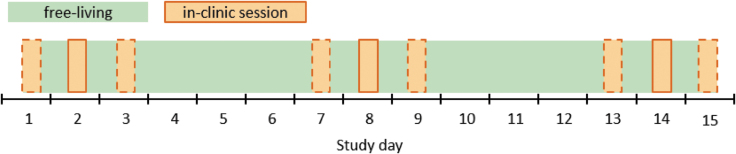
Schematic depiction of an example study protocol for a CGM system with a sensor lifetime of 14 days and three in-clinic sessions per participant. The dashed orange squares indicate other possible study days for in-clinic sessions, thus requiring suitable scheduling to cover study days 1, 2, 3, 7, 8, 9, 13, 14, and 15 equally often. CGM, continuous glucose monitoring.

We propose a study protocol in which the subjects spend the majority of the sensor lifetime in the free-living setting, interrupted by several in-clinic sessions lasting 8–12 h. In-clinic sessions should be scheduled so that their overall distribution equally reflects the beginning, middle, and end of the sensor lifetime, in accordance with both the POCT05 guideline and FDA iCGM requirements.^7,8^ This approach minimizes the burden on the subjects associated with the in-clinic session procedures. The exact distribution, as well as the number of sessions per subject, is dependent on the sensor lifetime. A possible study protocol for a CGM system with a 14-day lifetime is provided in Figure 1. It can easily be adapted for CGM systems with different sensor lifetimes.

### CGM system use

Regarding the CGM sensor insertion and calibration, we also refer to the POCT05 guideline.^7^ There it is recommended that, if applicable, the sensors should be inserted by the participants themselves under the supervision of the study staff and that all intended sensor locations, for example, arm and abdomen, are examined.

To assess between-sensor precision, the CGM data from two sensors worn simultaneously by the same participant should be compared. For that, it is necessary to insert two sensors at adjacent sites, at least for a subgroup of participants. However, we recommend including only one sensor per application site and participant (determined before insertion) in the main accuracy analysis because it likely affects the statistical independence of results. Here, a study with a factory-calibrated CGM system, where two sensors were worn simultaneously, estimated that the between-participant variance in sensor-specific mean absolute relative differences (MARDs) contributed 44% to the total variation in sensor-specific MARDs.^40^ This shows that the variation in characteristics between participants significantly contributes to the overall variance in accuracy. It is therefore possible that the inclusion of multiple sensors from the same participant would lead to an underestimation of variance in accuracy. Moreover, the CGM systems should be calibrated according to the minimally required schedule and we advise that calibrations should not be carried out during or immediately before the frequent sampling period (FSP) (see section “Sampling protocol and comparator measurements”).

## Free-**L**iving **T**esting **P**rocedures

In the free-living setting, we propose to ask participants to follow their regular daily routine and carry out at least seven capillary BG measurements per day with a BGM system fulfilling the accuracy requirements of the current ISO15197 standard,^41^ or the FDA over-the-counter guidance.^42^ The timing of these measurements should follow the established seven-point profile (immediately before and 2 h after breakfast, lunch, and dinner, and before bed).^43^

In addition, we recommend that the participants should perform the measurements in duplicate from the same finger prick. This allows the retrospective identification of outliers, for example, due to handling errors, and reduces imprecision as the results from duplicate measurements can be averaged. If considered feasible, participants may also be asked to perform a third measurement if the relative difference (with respect to the first measurement) of the duplicate exceeds ±10% if the first measurement is ≥100 mg/dL (5.6 mmol/L), or ±10 mg/dL (0.56 mmol/L) if the first measurement is <100 mg/dL (5.6 mmol/L).

## In-**C**linic **T**esting **P**rocedures and **C**omparator **D**ata **C**haracterization

### Sampling protocol and comparator measurements

We propose that the in-clinic sessions should include an FSP, where comparator measurements are carried out every 15 min over a period between 6 and 8 h. This duration agrees with the median FSP duration of 7.5 h, estimated in our recent literature review of CGM performance studies,^10^ and is adequate to produce comparator data with the recommended characteristics, as explained below. The 15-min sampling interval is in accordance with the POCT05 guideline,^7^ and is deemed sufficiently narrow to capture the variability of short-term BG concentration fluctuations and to estimate their RoC.^44^ In a number of recent studies,^19,20,30,45,46^ this sampling interval was shortened during phases of hypo- and hyperglycemia, presumably to increase safety and boost the number of comparator data points in these glycemic regions. However, as we have mentioned in a previous article,^10^ this approach has led to concerns about the statistical interdependency of the measurements. Furthermore, a constant sampling interval greatly facilitates the standardization of data analysis and statistical performance evaluation. We therefore recommend a protocol with a constant sampling interval of 15 min.

In our review on CGM performance, we found that comparator BG concentrations are measured in capillary, venous, and arterialized-venous blood.^10^ These approaches can yield different results due to physiologically different BG concentrations in these blood samples and thus affect the observed CGM performance. It is therefore crucial to standardize the comparator measurement procedure. However, a detailed discussion of this topic lies beyond the scope of this article and will be discussed in a future article. In principle, the in-clinic testing procedures suggested in this article are independent from the chosen approach. However, it should be mentioned that a safe and effective manipulation of BG concentrations, as described below, requires that measurement results are available in near real-time to decide on food intake and/or insulin dosing.

Irrespective of the chosen comparator measurement approach, the schedule for capillary measurements with the BGM system followed during free-living days should also be implemented on days with in-clinic sessions to provide capillary comparator measurements on every study day.

### The dynamic glucose region plot

Users of CGM systems routinely combine the displayed information on current sensor glucose levels and their accompanied RoC (in the form of trend arrows) to make therapeutic decisions. We therefore propose to evaluate the CGM performance under varying combinations of BG concentration and RoC, which can be illustrated as follows. The same RoC of −3 mg/(dL·min) [−0.17 mmol/(L·min), ↓↓] has a different clinical interpretation depending on whether the BG level is at 90 mg/dL (5 mmol/L) or 230 mg/dL (12.8 mmol/L). Likewise, the same BG level of 72 mg/dL (4 mmol/L) would lead to different therapeutic decisions depending on whether the RoC is +2 mg/(dL·min) [+0.11 mmol/(L·min), ↑] or −2 mg/(dL·min) [−0.11 mmol/(L·min), ↓].

**FIG. 2.**
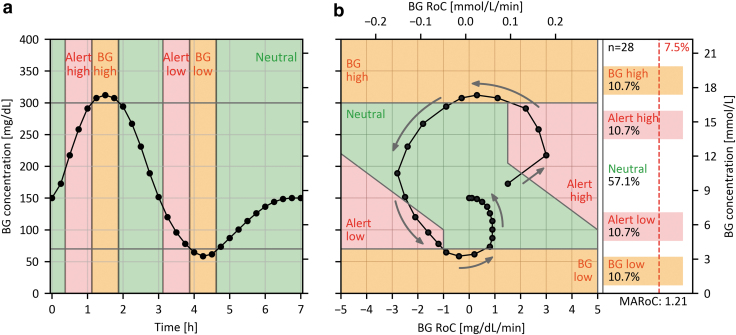
**(a)** Target profile of BG concentrations over time during an in-clinic session. **(b)** DGR plot showing the associated profile of combined RoC and BG. The gray arrows indicate the direction of the curve with respect to its evolution over time. Note that there is no RoC-BG pair for time zero, because each BG value is paired with an RoC calculated with respect to the preceding BG value. The colored background indicates the critical regions in which adequate CGM accuracy is of particular importance. The panel on the right shows the percentages of RoC-BG pairs in the respective regions, the proposed minimum required percentage for the critical regions (7.5%), the MARoC in mg/(dL**·**min), and the number of RoC-BG pairs (*n* = 28). BG, blood glucose; DGR, dynamic glucose region; MARoC, mean absolute RoC; RoC, rate of change.

We have therefore developed a graphical representation for the combination of BG concentrations and RoC, referred to as the dynamic glucose region (DGR) plot, based on similar representation called dynamic risk space.^47^ Figure 2 shows how a glucose profile obtained during an in-clinic session translates to the DGR plot.

As shown in Figure 2b, the area of the DGR plot is divided into five regions comprising four critical regions (red and orange) and one neutral region (green). The critical regions were chosen to reflect situations in which adequate CGM accuracy is of particular importance and are based on commonly used BG concentration thresholds.

The orange regions termed “BG high” and “BG low” acknowledge the need to test the CGM systems across their measurement range, consistent with existing guidelines.^7,8^ We chose BG concentrations <70 mg/dL (3.9 mmol/L) for the “BG low” region and >300 mg/dL (16.7 mmol/L) for the “BG high” region, independent of the associated RoCs. The lower threshold was chosen in deviation from the POCT05 guideline (<80 mg/dL [4.4 mmol/L]^7^) to align with the consensus threshold for level 1 hypoglycemia,^48,49^ and to be closer to the lower limit of the measurement range of most CGM systems (40 or 50 mg/dL [2.2 or 2.8 mmol/L]).

The upper threshold of 300 mg/dL (16.7 mmol/L) was chosen in accordance with POCT05 guideline but differing from the threshold for level 2 hyperglycemia (250 mg/dL [13.9 mmol/L])^50^ to be closer to the upper limit of the measurement range of most CGM systems of 400 mg/dL (22.2 mmol/L), giving this region a more technical, rather than a clinical, justification.

The two red regions in Figure 2b labeled “Alert low” and Alert high” represent situations with moderate-to-fast RoCs and impending hypo- or hyperglycemia. The “Alert low” region with rapidly falling BG concentrations in the normal range has the highest clinical risk of an adverse event, for example, severe hypoglycemia, and is therefore of particular importance. It is also the region in which AID systems typically suspend insulin infusion. This region represents BG levels ≥70 mg/dL (3.9 mmol/L) and RoCs < −1 mg/(dL·min) [−0.06 mmol/(L·min)]. Furthermore, the region includes RoC-BG combinations, that, given the current RoC, would lead to BG concentrations below 70 mg/dL (3.9 mmol/L) within 30 min, which causes the upper border of this region to be tilted. The time interval of 30 min was chosen as it is gives diabetes patients sufficient time to take action and it is used as the prediction horizon in some current-generation AID systems.^51,52^ RoCs between −1 mg/(dL·min) [−0.06 mmol/(L·min)] and 0 mg/(dL·min) were not included in this region to ensure that the CGM systems are tested at enough rapidly falling RoCs.

Accordingly, the “Alert high” region is defined by BG levels ≤300 mg/dL (16.7 mmol/L), RoCs > +1.5 mg/(dL·min) [+0.08 mmol/(L·min)], and current BG-RoC combinations leading to a BG level increase above 250 mg/dL (13.9 mmol/L, level 2 hyperglycemia) within the following 30 min.

The remaining RoC-BG combinations in the green region termed “Neutral” are less clinically relevant. Nevertheless, the majority of data points will be found in this region due to the practical nature of CGM performance studies. The question of how the collected data points should be distributed across the regions is addressed in the section “Recommendations for the comparator data distribution.” A summary of all region definitions is provided in Table 1.

**Table 1.** Definition of the Regions in the Dynamic Glucose Region Plot and Recommended Percentages of Rate of Change—Blood Glucose Data Points in Each Region

Region	Color	Definition	Recommended percentage, %
BG low	Orange	BG <70 mg/dL (3.9 mmol/L)	≥7.5
		Any RoC	
BG high	Orange	BG >300 mg/dL (16.7 mmol/L)	≥7.5
		Any RoC	
Alert low	Red	BG ≥70 mg/dL (3.9 mmol/L)	≥7.5
		RoC < −1 mg/(dL·min) [−0.06 mmol/(L·min)]	
		BG <70 mg/dL (3.9 mmol/L) within 30 min at current RoC	
Alert high	Red	BG ≤300 mg/dL (16.7 mmol/L)	≥7.5
		RoC > +1.5 mg/(dL·min) [+0.08 mmol/(L·min)]	
		BG >250 mg/dL (13.9 mmol/L) within 30 min at current RoC	
Neutral	Green	All other RoC-BG pairs^a^	≤70

^a^ BG levels should be paired with RoC values calculated from the current and preceding BG level.

BG, blood glucose; RoC, rate of change.

### Analysis of real-world CGM data

To demonstrate the need to create a wide range of BG concentrations and RoCs during the in-clinic sessions as well as to legitimize the critical regions of the DGR plot, real-world CGM data collected in five different studies,^4,53–56^ made publicly available,^57^ were pooled and analyzed. The pooled data set contains more than 4.3 million hours of CGM data from 897 individuals with type 1 diabetes between the ages of 2 and 86 years. Additional details on the data sets and data analysis are provided in the Supplementary Data.

**FIG. 3.**
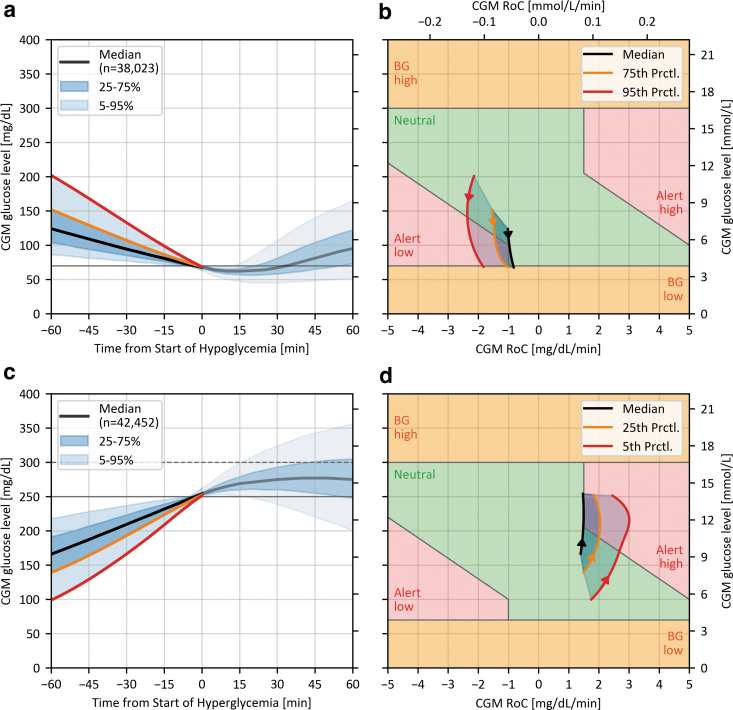
Results from the analysis of real-world CGM data concerning the “Alert low” **(a, b)** and “Alert high” **(c, d)** region. **(a, c)** Show the summary statistics of CGM traces from −60 to +60 min after the start of a hypo-/hyperglycemic episode according to the recommendations of the Ambulatory Glucose Profile.^59^ **(b, d)** Show the interpolated and smoothed CGM RoC—glucose-level races of selected summary statistics from −60 to the start of the hypo-/hyperglycemic episodes. The arrows indicate the direction of the curves over time.

To validate the boundaries of the “Alert low” region, CGM traces recorded in the 60 min before hypoglycemic episodes were analyzed (Fig. 3a, b). A hypoglycemic episode was included in the analysis if consecutive CGM glucose levels were <70 mg/dL (3.9 mmol/L) for at least 15 min,^58^ and up to 120 min. In addition, for any episode to be included in the analysis, CGM glucose levels had to be ≥70 mg/dL (3.9 mmol/L) and monotonically decreasing (RoC ≤0) in the 60 min before the episode of interest. This led to the selection of more than 38,000 CGM traces containing a hypoglycemic episode. Considering only the steeper descents into hypoglycemia because they carry a higher risk for an adverse event, shown in Figure 3b, it is demonstrated that ∼50% of prehypoglycemia CGM traces traverse the “Alert low” region. This justifies the need to expose the CGM systems to those combinations of BG concentrations and RoCs during performance testing.

Similar relevance can be assigned to the “Alert high” region (Fig. 3c, d) with a corresponding analysis. A hyperglycemic episode was defined as CGM glucose levels >250 mg/dL (13.9 mmol/L) for at least 15 min,^58^ and for any episode to be included in the analysis, CGM glucose levels had to be ≤250 mg/dL (13.9 mmol/L) and monotonically increasing (RoC ≥0) in the 60 min before any episode. This led to the selection of more than 42,000 CGM traces. As shown in Figure 3c, ∼25% of selected CGM traces eventually crossed the 300 mg/dL (16.7 mmol/L) threshold (dotted line in Fig. 3c), giving strong justification to the “BG high” region.

### Glucose manipulation procedures

The following section introduces a possible glucose manipulation procedure designed to generate comparator data in the proposed regions of the DGR plot in a safe, ethical, and consistent manner. We want to emphasize that other testing procedures adequately covering the critical regions in the DGR plot are possible. The description of the procedure provided below focuses on the most important aspects and is not intended to be an exhaustive protocol to be used as a template.

The basis of our suggestion for the in-clinic testing procedures will form the target glucose profile (TGP), shown in Figure 2a. The TGP was designed to traverse all regions of the DGR plot within a single in-clinic session and a 7-h FSP. The TGP includes distinct and transient hyper- and hypoglycemic episodes similar to the ones observed in the real-life data set (Fig. 3). This allows a more realistic assessment of alert reliability in comparison with testing procedures that induce hypo- and hyperglycemic episodes lasting 60 min or longer. The general approach suggested by the TGP, that is, inducing hyperglycemia followed by hypoglycemia, is similar to a number of approaches reported in the literature,^13,14,23–32^ as discussed above. Using the reverse order, that is, to start with hypoglycemia followed by hyperglycemia, might be a suitable approach to populate the “BG low,” “Alert high,” and “BG high” regions. However, when the subjects' BG levels are lowered toward hypoglycemia at the start of an in-clinic session from fasting conditions, it might be difficult to produce the rapidly falling BG concentrations that are required to populate the “Alert low” region, whose clinical relevance has been shown in the analysis of real-world data.

The procedure described below is most suitable for participants with type 1 diabetes because it relies on the adjustment of timing and dosing of rapidly acting insulin. In addition, participants should have well-established insulin correction and carbohydrate-to-insulin factors and no relevant comorbidities (in particular gastroparesis). BG concentrations should be closely monitored throughout the in-clinic session and additional measurements outside the previously recommended 15-min interval may be carried out to ensure participant safety but should be excluded from data analysis. In addition, we recommend that this procedure is only carried out by study sites experienced in the management of people with diabetes.

Before the start of the FSP, the participant's BG concentrations should be stable within the target range (70–180 mg/dL [3.9–10 mmol/L]) and any AID systems should be switched to manual mode, that is, no automated adaptation of insulin delivery, until the end of the FSP. At the start of the FSP, the participants are served a meal specifically selected for this procedure. We suggest a meal containing a maximum of ∼25% of the participants' daily caloric demand consisting of 65% carbohydrates (both slow and fast absorbed), 20% fat, and 15% protein. The bolus of short-acting insulin that would normally be delivered at or around mealtime is then withheld until BG levels have reached values between 250 and 300 mg/dL (13.9 and 16.7 mmol/L). This should induce the glycemic conditions indicative of the “Alert high” and “BG high” regions, that is, a rapid BG concentration rise as well as sustained BG levels >300 mg/dL (16.7 mmol/L) for ∼30 min. The size of the bolus should be calculated based on the participant-specific therapy parameters, the carbohydrate content of the meal, and the BG level at the start of FSP with a target BG level of 100 mg/dL (5.6 mmol/L). A general increase in the bolus size to induce the subsequent hypoglycemia is typically not necessary.

Our experience indicates that, after reaching their peak, BG levels drop rapidly toward hypoglycemia traversing the “Alert low” and “BG low” regions. Should this not happen, additional insulin boluses can be delivered. Once BG concentrations below 70 mg/dL (3.9 mmol/L) have been reached or are imminent, appropriate hypoglycemia interventions are implemented. The specific amount, composition, and timing of food intake for this intervention should be decided at the discretion of study staff in agreement with the participant. However, we suggest to choose the hypoglycemia intervention strategy such that BG concentrations are maintained <70 mg/dL (3.9 mmol/L) for ∼30 min, while avoiding rebound hyperglycemia through excessive carbohydrate intake. For the remainder of the FSP, the BG levels should be kept stable in the range 100–180 mg/dL (5.5–10 mmol/L), allowing a safe release of the participants at the end of the FSP.

We suggest that any further meals or snacks are low in carbohydrate content and that the remaining insulin action of the first bolus is considered in any decisions on insulin dosing/timing. If the participants' BG levels should not respond as expected, and not all critical regions of the DGR plot are reached, corrective actions can be taken as long as they are deemed safe by the study staff. These actions can include additional insulin boluses, the adaptation of basal insulin infusion for participants using insulin pumps, food intake, or mild exercise.

**FIG. 4.**
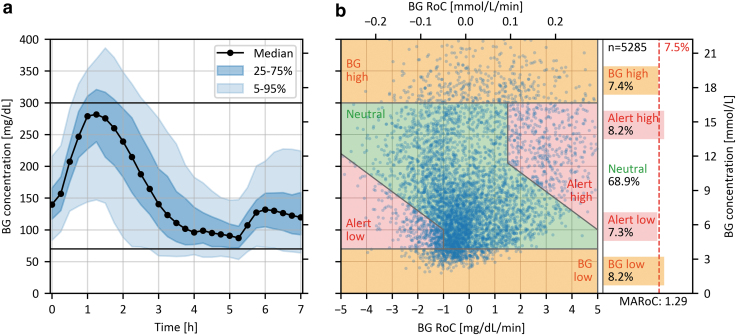
Characteristics of comparator data collected in a study with 50 participants during 194 in-clinic sessions. **(a)** Summary statistics of the time profile of BG concentrations. **(b)** DGR plot showing the individual RoC-BG pairs. The area on the right shows the percentage of RoC-BG pairs in the respective regions, the proposed minimum required percentage for the critical regions (7.5%), the total number of RoC-BG pairs (*n* = 5285), and the MARoC in mg/(dL·min) at the bottom.

The characteristics of the comparator data collected in a study with 50 participants and 195 in-clinic sessions implementing a similar testing procedure are shown in Figure 4. The main differences between the protocol followed in this study and the procedure described in this article are the use of a slightly smaller meal at the beginning of the FSP and the consumption of a freely chosen second meal after 5.5 h. Furthermore, 11 (22%) participants underwent no manipulation of insulin dosing and followed their routine therapy after consuming the test meal. This explains the lower initial BG peak and the sharp rise after 5.5 h in comparison with the TGP (Fig. 4a). This figure also demonstrates the variability in profiles regarding the time of hypoglycemia, despite applying the same procedure in every participant and in-clinic session.

Figure 4b shows that ∼31% of all RoC-BG pairs were in the critical regions and that the “Alert low” and “BG high” regions were slightly less represented than the other critical regions.

### Recommendations for comparator data distribution

After defining the critical regions in the DGR plot and describing a possible glucose manipulation procedure, we can introduce our recommendation for the characteristics of the comparator data distribution; similar but less stringent in comparison with the ISO 15197:2015 standard for BGM systems.^41^ Defining the characteristics of the comparator data is more practicable than mandating a particular testing procedure in a standardized study design.

The TGP (Fig. 2) and associated procedure were designed so that ideally, ∼11% of data points would be found in each critical region, leaving around 57% in the neutral region. However, due to the expected natural variability in the comparator data, exemplified in Figure 4, it is unlikely that this ideal distribution is achieved. Furthermore, it should not be necessary to apply the glucose manipulation procedure to all participants included in the study, thus allowing the possibility to include subjects with type 2 diabetes as mentioned above. We propose that the minimal percentage of data points lying in each critical region should be 7.5%, leaving at most 70% of data points in the neutral region. Note that these recommendations should be fulfilled for only the comparator data points that are used for the CGM accuracy evaluation, that is, after they have been paired with the CGM data. A proposal for the absolute number of data points to be found in each region is beyond the scope of this article as these numbers depend on the chosen performance parameters and possible minimum acceptance criteria for those parameters.

Should it be anticipated that the proposed recommendation of 7.5% of data points in each critical region is not fulfilled, it is possible for some participants to follow a glucose manipulation procedure that fills specific regions of the DGR plot. For example, if additional hypoglycemic BG concentrations are required, it is possible to implement the previously mentioned approach of starting with hypoglycemia followed by hyperglycemia. Alternatively, we propose the following procedure for discarding the least critical RoC-BG pairs from the neutral region until the recommended percentages are achieved. This procedure is exemplified on the data set shown in Figure 4, where the “BG high” and “Alert low” regions have an insufficient number of data points.

For each RoC-BG pair, a Euclidian distance measure *D* with respect to the center of the “neutral” region, defined at a BG level of 185 mg/dL (10.3 mmol/L) and an RoC of 0, is calculated as follows:

$$D=\sqrt{{\left(\frac{RoC}{a}\right)}^{2}+{\left(\frac{BG-BG_{c}}{b}\right)}^{2}},$$

where the value for *a* is set to 1 mg/(dL·min) [0.06 mmol/(L·min)], the value for *b* is set to 115 mg/dL (6.4 mmol/L), and BG_*c*_ represents the center of the neutral region set to 185 mg/dL (10.3 mmol/L). Subsequently, the RoC-BG pairs with the lowest values of *D* are successively excluded until the recommended minimal percentages for all critical regions are met.

**FIG. 5.**
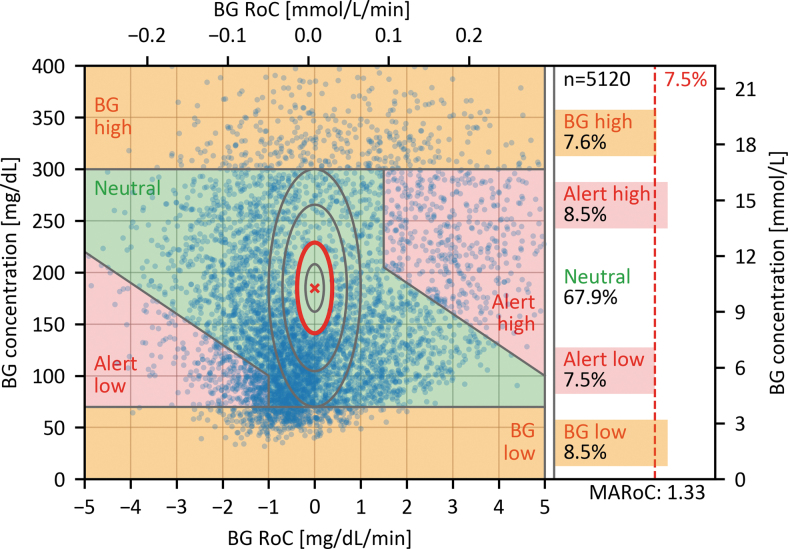
DGR plot exemplifying the procedure for eliminating data from the neutral region using the data presented in Figure 4. The defined center of the neutral region is marked with a red cross, and the minimal elliptic exclusion region to achieve the desired distribution of at least 7.5% of comparator data in each critical region is outlined in red. The gray concentric ellipses have the same height-to-width ratio and indicate other possible regions for elimination of data. The shape of the ellipse was chosen to stretch between −1 and +1 mg/(dL·min) [−0.06 and +0.06 mmol/(L·min)] at the horizontal edges of the neutral region (70 mg/dL [3.9 mmol/L] and 300 mg/dL [16.7 mmol/L]).

The geometric interpretation of this procedure is exemplified in Figure 5 and shows that the elimination region takes the form of an ellipse with a constant height-to-width ratio. The shape of the ellipse, determined by the values for *a* and *b*, was chosen to stretch between −1 and +1 mg/(dL·min) [−0.06 and +0.06 mmol/(L·min)] at the horizontal edges of the neutral region (70 mg/dL [3.9 mmol/L] and 300 mg/dL [16.7 mmol/L]), in which case *D* has a value of 1. The size of the ellipse is roughly determined by the number of data points that need to be excluded but it should not stretch into the “BG high” and “BG low” region. This means that, if any RoC-BG pairs with a *D* > 1 have to be excluded, the underlying data set may be inadequate to comply with the recommended distribution of comparator data.

This procedure for eliminating data was designed to be easily adopted, independent from the corresponding CGM data and without the need for random sampling, therefore allowing reproducibility and facilitating standardization. For the example data set in Figure 5, a total of 165 data points, corresponding to 3.1% of all data pairs, had to be excluded for the percentage of data points in all the critical regions to be at least 7.5%. This demonstrates that a study implementing a testing procedure similar to the one described in this article can fulfill our recommendation with a justifiable number of excluded data points, even if only 78% of participants undergo the full glucose manipulation procedure.

## Conclusion

This article, for the first time, provides a comprehensive proposal and justification for the distribution of comparator BG concentrations and RoCs to be produced during CGM performance testing. Our proposal was built upon results from a previous study and utilizes the newly developed DGR plot identifying clinically relevant combinations of glucose levels and their RoC. We encourage researchers to use this tool when reporting results of CGM performance studies, thus increasing transparency and facilitating the comparison of results between studies.^10^ A free and open-source software package to create the DGR plot is published with this article (https://github.com/IfDTUlm/CGM_Performance_Assessment).

We also suggest the basic elements for the development of a study protocol and in-clinic testing procedures capable of fulfilling the proposed characteristics of comparator data. Implementing a similar procedure in future studies would harmonize the comparator data characteristics across studies. As a result, the observed performance of CGM systems is expected to be independent from the study design and becomes comparable, even when tested in separate studies. We expect that this work is an important element of a future standard for the performance evaluation of CGM systems.

A pilot verification study is planned based on our proposal, and we would like to invite other research centers and manufacturers of CGM systems to test the feasibility of the proposed comparator data distribution and testing procedures to establish their suitability for incorporation in an internationally recognized standard for the clinical performance evaluation of CGM systems.

## Supplementary Material

Supplemental data

## References

[1] Elbalshy M, Haszard J, Smith H, et al. Effect of divergent continuous glucose monitoring technologies on glycaemic control in type 1 diabetes mellitus: A systematic review and meta-analysis of randomised controlled trials. Diabet Med 2022;39(8):e14854; doi: 10.1111/dme.14854 35441743 PMC9542260

[2] Holt RIG, DeVries JH, Hess-Fischl A, et al. The management of type 1 diabetes in adults. A consensus report by the American Diabetes Association (ADA) and the European Association for the Study of Diabetes (EASD). Diabetologia 2021;64(12):2609–2652; doi: 10.1007/s00125-021-05568-3 34590174 PMC8481000

[3] ElSayed NA, Aleppo G, Aroda VR, et al. 7. Diabetes technology: Standards of care in diabetes—2023. Diabetes Care 2022;46(Suppl 1):S111–S127; doi: 10.2337/dc23-S007 PMC9810474 36507635

[4] Brown SA, Kovatchev BP, Raghinaru D, et al. Six-month randomized, multicenter trial of closed-loop control in type 1 diabetes. N Engl J Med 2019;381(18):1707–1717; doi: 10.1056/NEJMoa1907863 31618560 PMC7076915

[5] Pemberton JS, Wilmot EG, Barnard-Kelly K, et al. CGM accuracy: Contrasting CE marking with the governmental controls of the USA (FDA) and Australia (TGA): A narrative review. Diabetes Obes Metab 2023;25(4):916–939; doi: 10.1111/dom.14962 36585365

[6] Clinical and Laboratory Standards Institute (CLSI). Performance Metrics for Continuous Interstitial Glucose Monitoring; Approved Guideline. CLSI Document POCT05-A. Clinical and Laboratory Standards Institute:2008.

[7] Clinical and Laboratory Standards Institute (CLSI). Performance Metrics for Continuous Interstitial Glucose Monitoring, 2nd ed. CLSI Guideline POCT05. Clinical and Laboratory Standards Institute: 2020.

[8] U.S. Food and Drug Administration. Evaluation of Automatic Class III Designation for Dexcom G6 Continuous Glucose Monitoring System: Decision Summary; 2018. Available from: https://www.accessdata.fda.gov/cdrh_docs/reviews/DEN170088.pdf [Last accessed: May 24, 2022].

[9] Heinemann L, Schoemaker M, Schmelzeisen-Redecker G, et al. Benefits and limitations of MARD as a performance parameter for continuous glucose monitoring in the interstitial space. J Diabetes Sci Technol 2020;14(1):135–150; doi: 10.1177/1932296819855670 31216870 PMC7189145

[10] Freckmann G, Eichenlaub M, Waldenmaier D, et al. Clinical performance evaluation of continuous glucose monitoring systems: A scoping review and recommendations for reporting. J Diabetes Sci Technol 2023;17(6):1506–1526; doi: 10.1177/19322968231190941 37599389 PMC10658695

[11] Freckmann G, Nichols JH, Hinzmann R, et al. Standardization process of continuous glucose monitoring: Traceability and performance. Clin Chim Acta 2021;515:5–12; doi: 10.1016/j.cca.2020.12.025 33359497

[12] Pleus S, Schoemaker M, Morgenstern K, et al. Rate-of-change dependence of the performance of two CGM systems during induced glucose swings. J Diabetes Sci Technol 2015;9(4):801–807; doi: 10.1177/1932296815578716 25852074 PMC4525645

[13] Kropff J, van Steen SC, deGraaff P, et al. Venous, arterialized-venous, or capillary glucose reference measurements for the accuracy assessment of a continuous glucose monitoring system. Diabetes Technol Ther 2017;19(11):609–617; doi: 10.1089/dia.2017.0189 28829160

[14] Boscari F, Galasso S, Facchinetti A, et al. FreeStyle Libre and Dexcom G4 Platinum sensors: Accuracy comparisons during two weeks of home use and use during experimentally induced glucose excursions. Nutrition, metabolism, and cardiovascular diseases. Nutr Metab Cardiovasc Dis 2018;28(2):180–186; doi: 10.1016/j.numecd.2017.10.023 29258716

[15] Link M, Kamecke U, Waldenmaier D, et al. Comparative accuracy analysis of a real-time and an intermittent-scanning continuous glucose monitoring system. J Diabetes Sci Technol 2021;15(2):287–293; doi: 10.1177/1932296819895022 31847555 PMC8256076

[16] Fokkert MJ, van Dijk PR, Edens MA, et al. Performance of the FreeStyle Libre Flash glucose monitoring system in patients with type 1 and 2 diabetes mellitus. BMJ Open Diab Res Care 2017;5(1):e000320; doi: 10.1136/bmjdrc-2016-000320 PMC5316912 28243449

[17] International Federation of Clinical Chemistry and Laboratory Medicine Scientific Division. Working Group on Continuous Glucose Monitoring (WG-CGM). 2019. Available from: https://ifcc.org/ifcc-scientific-division/sd-working-groups/wg-cgm/ [Last accessed: July 4, 2023].

[18] Wadwa RP, Laffel LM, Shah VN, et al. Accuracy of a factory-calibrated, real-time continuous glucose monitoring system during 10 days of use in youth and adults with diabetes. Diabetes Technol Ther 2018;20(6):395–402; doi: 10.1089/dia.2018.0150 29901421 PMC6110124

[19] Alva S, Bailey T, Brazg R, et al. Accuracy of a 14-day factory-calibrated continuous glucose monitoring system with advanced algorithm in pediatric and adult population with diabetes. J Diabetes Sci Technol 2022;16(1):70–77; doi: 10.1177/1932296820958754 32954812 PMC8875061

[20] Garg SK, Liljenquist D, Bode B, et al. Evaluation of accuracy and safety of the next-generation up to 180-day long-term implantable eversense continuous glucose monitoring system: The PROMISE study. Diabetes Technol Ther 2022;24(2):84–92; doi: 10.1089/dia.2021.0182 34515521 PMC8817689

[21] The Diabetes Research in Children Network (DIRECNET) Study Group. The accuracy of the GlucoWatch G2 biographer in children with type 1 diabetes: results of the diabetes research in children network (DirecNet) accuracy study. Diabetes Technol Ther 2003;5(5):791–800; doi: 10.1089/152091503322526996 14633344 PMC2302793

[22] Weinstein RL, Schwartz SL, Brazg RL, et al. Accuracy of the 5-day FreeStyle navigator continuous glucose monitoring system: Comparison with frequent laboratory reference measurements. Diabetes Care 2007;30(5):1125–1130; doi: 10.2337/dc06-1602 17337488

[23] Wentholt IM, Vollebregt MA, Hart AA, et al. Comparison of a needle-type and a microdialysis continuous glucose monitor in type 1 diabetic patients. Diabetes Care 2005;28(12):2871–2876; doi: 10.2337/diacare.28.12.2871 16306547

[24] Wilhelm B, Forst S, Weber MM, et al. Evaluation of CGMS during rapid blood glucose changes in patients with type 1 diabetes. Diabetes Technol Ther 2006;8(2):146–155; doi: 10.1089/dia.2006.8.146 16734545

[25] Freckmann G, Pleus S, Link M, et al. Performance evaluation of three continuous glucose monitoring systems: Comparison of six sensors per subject in parallel. J Diabetes Sci Technol 2013;7(4):842–853; doi: 10.1177/193229681300700406 23911165 PMC3879748

[26] Luijf YM, Mader JK, Doll W, et al. Accuracy and reliability of continuous glucose monitoring systems: A head-to-head comparison. Diabetes Technol Ther 2013;15(8):722–727; doi: 10.1089/dia.2013.0049 23650900 PMC3746288

[27] Zschornack E, Schmid C, Pleus S, et al. Evaluation of the performance of a novel system for continuous glucose monitoring. J Diabetes Sci Technol 2013;7(4):815–823; doi: 10.1177/193229681300700403 23911162 PMC3879745

[28] Kropff J, Bruttomesso D, Doll W, et al. Accuracy of two continuous glucose monitoring systems: A head-to-head comparison under clinical research centre and daily life conditions. Diabetes Obes Metab 2015;17(4):343–349; doi: 10.1111/dom.12378 25132320 PMC4409843

[29] Freckmann G, Link M, Pleus S, et al. Measurement performance of two continuous tissue glucose monitoring systems intended for replacement of blood glucose monitoring. Diabetes Technol Ther 2018;20(8):541–549; doi: 10.1089/dia.2018.0105 30067410 PMC6080122

[30] Boscari F, Vettoretti M, Amato AML, et al. Comparing the accuracy of transcutaneous sensor and 90-day implantable glucose sensor. Nutr Metab Cardiovasc Dis 2021;31(2):650–657; doi: 10.1016/j.numecd.2020.09.006 33594987

[31] Pleus S, Stuhr A, Link M, et al. Variation of mean absolute relative differences of continuous glucose monitoring systems throughout the day. J Diabetes Sci Technol 2022;16(3):649–658; doi: 10.1177/1932296821992373 33615834 PMC9294578

[32] Hochfellner DA, Simic A, Taucher MT, et al. Accuracy assessment of the GlucoMen^®^ day CGM system in individuals with type 1 diabetes: A pilot study. Biosensors 2022;12(2):106; doi: 10.3390/bios12020106 35200366 PMC8869704

[33] Keenan DB, Mastrototaro JJ, Zisser H, et al. Accuracy of the Enlite 6-day glucose sensor with guardian and Veo calibration algorithms. Diabetes Technol Ther 2012;14(3):225–231; doi: 10.1089/dia.2011.0199 22145851

[34] Feldman B, Brazg R, Schwartz S, et al. A continuous glucose sensor based on wired enzyme technology—Results from a 3-day trial in patients with type 1 diabetes. Diabetes Technol Ther 2003;5(5):769–779; doi: 10.1089/152091503322526978 14633342

[35] Peoples T, Bailey T, Ronald B, et al. Accuracy performance of the Medtronic NexSensor^TM^ for 6 days in an inpatient setting using abdomen and buttocks insertion sites. J Diabetes Sci Technol 2011;5(2):358–364; doi: 10.1177/193229681100500224 21527106 PMC3125929

[36] Zisser HC, Bailey TS, Schwartz S, et al. Accuracy of the SEVEN continuous glucose monitoring system: Comparison with frequently sampled venous glucose measurements. J Diabetes Sci Technol 2009;3(5):1146–1154; doi: 10.1177/193229680900300519 20144429 PMC2769895

[37] Christiansen M, Bailey T, Watkins E, et al. A new-generation continuous glucose monitoring system: Improved accuracy and reliability compared with a previous-generation system. Diabetes Technol Ther 2013;15(10):881–888; doi: 10.1089/dia.2013.0077 23777402 PMC3781114

[38] Bailey TS, Chang A, Christiansen M. Clinical accuracy of a continuous glucose monitoring system with an advanced algorithm. J Diabetes Sci Technol 2015;9(2):209–214; doi: 10.1177/1932296814559746 25370149 PMC4604574

[39] Mazze RS, Strock E, Borgman S, et al. Evaluating the accuracy, reliability, and clinical applicability of continuous glucose monitoring (CGM): Is CGM ready for real time? Diabetes Technol Ther 2009;11(1):11–18; doi: 10.1089/dia.2008.0041 19132850

[40] Bailey T, Bode BW, Christiansen MP, et al. The performance and usability of a factory-calibrated flash glucose monitoring system. Diabetes Technol Ther 2015;17(11):787–794; doi: 10.1089/dia.2014.0378 26171659 PMC4649725

[41] International Organization for Standardization. In Vitro Diagnostic Test Systems—Requirements for Blood-Glucose Monitoring Systems for Self-Testing in Managing Diabetes Mellitus (ISO 15197:2013). 2015; EN ISO 15197:2015.

[42] U.S. Food and Drug Administration Center for Devices and Radiological Health. Self-Monitoring Blood Glucose Test Systems for Over-the-Counter Use: Guidance for Industry and Food and Drug Administration Staff. Silver Spring, MD, USA; 2020.

[43] Pleus S, Freckmann G, Schauer S, et al. Self-monitoring of blood glucose as an integral part in the management of people with type 2 diabetes mellitus. Diabetes Ther 2022;13(5):829–846; doi: 10.1007/s13300-022-01254-8 35416589 PMC9076772

[44] Clarke WL. The original Clarke Error Grid Analysis (EGA). Diabetes Technol Ther 2005;7(5):776–779; doi: 10.1089/dia.2005.7.776 16241881

[45] Garg SK, Kipnes M, Castorino K, et al. Accuracy and safety of Dexcom G7 continuous glucose monitoring in adults with diabetes. Diabetes Technol Ther 2022;24(6):373–380; doi: 10.1089/dia.2022.0011 35157505 PMC9208857

[46] Laffel LM, Bailey TS, Christiansen MP, et al. Accuracy of a seventh-generation continuous glucose monitoring system in children and adolescents with type 1 diabetes. J Diabetes Sci Technol 2023;17(4):962–967; doi: 10.1177/19322968221091816 35466707 PMC10347986

[47] Guerra S, Sparacino G, Facchinetti A, et al. A dynamic risk measure from continuous glucose monitoring data. Diabetes Technol Ther 2011;13(8):843–852; doi: 10.1089/dia.2011.0006 21561370

[48] American Diabetes Association Workgroup on Hypoglycemia. Defining and reporting hypoglycemia in diabetes: A report from the American Diabetes Association Workgroup on Hypoglycemia. Diabetes Care 2005;28(5):1245–1249; doi: 10.2337/diacare.28.5.1245 15855602

[49] International Hypoglycaemia Study Group. Glucose concentrations of less than 3.0 mmol/L (54 mg/dL) should be reported in clinical trials: A Joint Position Statement of the American Diabetes Association and the European Association for the Study of Diabetes. Diabetes Care 2017;40(1):155; doi: 10.2337/dc16-2215 27872155

[50] Battelino T, Danne T, Bergenstal RM, et al. Clinical targets for continuous glucose monitoring data interpretation: Recommendations from the international consensus on time in range. Diabetes Care 2019;42(8):1593–1603; doi: 10.2337/dci19-0028 31177185 PMC6973648

[51] Medtronic MiniMed. MiniMed™ 780G with the Guardian™ 4 Sensor System User Guide. RF: M994838A001. Northridge, CA, USA; 2023.

[52] Tandem Diabetes Care. T:Slim X2 Insulin Pump with Control-IQ Technology User Guide. 1005627_D. San Diego, CA, USA; 2022.

[53] Aleppo G, Ruedy KJ, Riddlesworth TD, et al. REPLACE-BG: A randomized trial comparing continuous glucose monitoring with and without routine blood glucose monitoring in adults with well-controlled type 1 diabetes. Diabetes Care 2017;40(4):538–545; doi: 10.2337/dc16-2482 28209654 PMC5864100

[54] Laffel LM, Kanapka LG, Beck RW, et al. Effect of continuous glucose monitoring on glycemic control in adolescents and young adults with type 1 diabetes: A randomized clinical trial. JAMA 2020;323(23):2388–2396; doi: 10.1001/jama.2020.6940 32543683 PMC7298603

[55] Pratley RE, Kanapka LG, Rickels MR, et al. Effect of continuous glucose monitoring on hypoglycemia in older adults with type 1 diabetes: A randomized clinical trial. JAMA 2020;323(23):2397–2406; doi: 10.1001/jama.2020.6928 32543682 PMC7298607

[56] Strategies to Enhance New CGM Use in Early Childhood (SENCE) Study Group. A randomized clinical trial assessing continuous glucose monitoring (CGM) use with standardized education with or without a family behavioral intervention compared with Fingerstick blood glucose monitoring in very young children with type 1 diabetes. Diabetes Care 2021;44(2):464–472; doi: 10.2337/dc20-1060 33334807 PMC9162100

[57] Jaeb Center for Health Research. Diabetes Datasets and Documents; 2023. Available from: https://public.jaeb.org/datasets/diabetes [Last accessed: April 19, 2023].

[58] Danne T, Nimri R, Battelino T, et al. International consensus on use of continuous glucose monitoring. Diabetes Care 2017;40(12):1631–1640; doi: 10.2337/dc17-1600 29162583 PMC6467165

[59] Bergenstal RM, Ahmann AJ, Bailey T, et al. Recommendations for standardizing glucose reporting and analysis to optimize clinical decision making in diabetes: The Ambulatory Glucose Profile (AGP). Diabetes Technol Ther 2013;15(3):198–211; doi: 10.1089/dia.2013.0051 23448694

