# Supplementary material for: Comparator Data Characteristics and Testing Procedures for the Clinical Performance Evaluation of Continuous Glucose Monitoring Systems
Source: Diabetes Technol Ther. 2024 Mar 28;26(4):263–75. doi: 10.1089/dia.2023.0465 (PMC10979680; doi:10.1089/dia.2023.0465)
Supplement: Supplemental data [file Supplementary_Data.pdf]

Supplemental Materials

Additional information on the analysis of real-world data

Studies and datasets

Table S 1: Detailed information on the publicly available datasets used in the article.

	REPLACE-BG[52,56]	***SENCE***[55,56]	***CITY***[53,56]	***WISDM***[54,56]	***DCLP3***[4,56]	***All***
**Number of participants** with CGM data	226	144	153	206	168	897
**Age** [years] Median (IQD*) [Min, Max]	43 (24) [19,78]	5 (3) [2,7]	17 (5) [14,24]	67 (7) [60,86]	28 (28) [14,71]	31 (45) [2,86]
**HbA1c** at screening [%] Median (IQD*) [Min, Max]	7.3 (1.0) [5.2,8.9]	8.2 (1.1) [7.0,9.9]	9.1 (1.7) [7.5,10.9]	7.5 (1.3) [5.4,10.0]	7.5 (1.2) [5.5,11.3]	7.8 (1.4) [5.2,11.3]
**Total duration** of CGM data [days]	51414.7	30152.0	28942.4	37477.9	31361.9	179349.0
**Duration** of CGM data per participant [days] Median (IQD*) [Min, Max]	230.6 (33.2) [56.5,326.7]	200.7 (165.9) [7.8,466.2]	184.6 (133.0) [7.6,381.7]	177.6 (103.1) [12.5,425.5]	189.6 (10.1) [125.8,200.5]	194.2 (69.0) [7.6,466.2]
**CGM system**	Dexcom G4 505	Dexcom G5 Mobile	Dexcom G5	Dexcom G5	Dexcom G6	-

* IQD: interquartile distance, i.e., difference between 75th and 25th percentiles

Data processing

The data recording interval was 5 minutes in all datasets and CGM values were provided as integers in mg/dL. Data from each subject was analyzed separately. To identify hypoglycemia episodes of interest, the following procedure was applied:

1. Identify all phases, where between 3 (15 min) and 24 (120 min) consecutive CGM glucose levels are <70 mg/dL (3.9 mmol/L). Any phases longer than 120 min are likely caused by artifacts and thus excluded.
2. Extend the phases identified in step 1 with the 12 preceding data points (60 min), forming the potential hypoglycemic episodes of interest.
3. Discard any potential hypoglycemic episodes of interest if:
   a. Any recording interval was not 5 minutes.

   b. The absolute value of any CGM rate of change (RoC) was >5 mg/dL/min (0.28 mmol/L/min).
   c. The preceding data contained CGM glucose levels <70 mg/dL (3.9 mmol/L).
   d. The preceding data contained CGM RoCs >0.

To identify hyperglycemia episodes of interest, the following procedure was applied:

1. Identify all phases, where 3 (15 min) or more consecutive CGM glucose levels are >250 mg/dL (13.9 mmol/L).
2. Extended the phases identified in step 1 with the 12 preceding data points (60 min), forming the potential hyperglycemic episodes of interest.
3. Discard any potential hyperglycemic episodes of interest if:
   a. Any recording interval was not 5 minutes.
   b. The absolute value of any CGM rate of change (RoC) was >5 mg/dL/min (0.28 mmol/L/min).
   c. The preceding data contained CGM glucose levels >250 mg/dL (13.9 mmol/L)
   d. The preceding data contained CGM RoCs <0.

The CGM RoC-glucose level traces shown in Figure 3b and 3d in the main text were calculated as follows:

1. Take the 13 consecutive CGM data points (60 min) including the first hypo-/hyperglycemic data point.
2. Fit a univariate spline function of degree 3 and smoothing factor 2 using the Python package *scipy.interpolate.UnivariateSpline.*
3. Use the fitted spline function to calculate an interpolated CGM trace of length 101.
4. Calculate the numeric derivative of the interpolated CGM trace to obtain the corresponding RoC curve and discard the first value of the interpolated CGM trace.

Additional results

The results of analyzing the individual datasets analogous to Figure 3 in the main text are shown below.

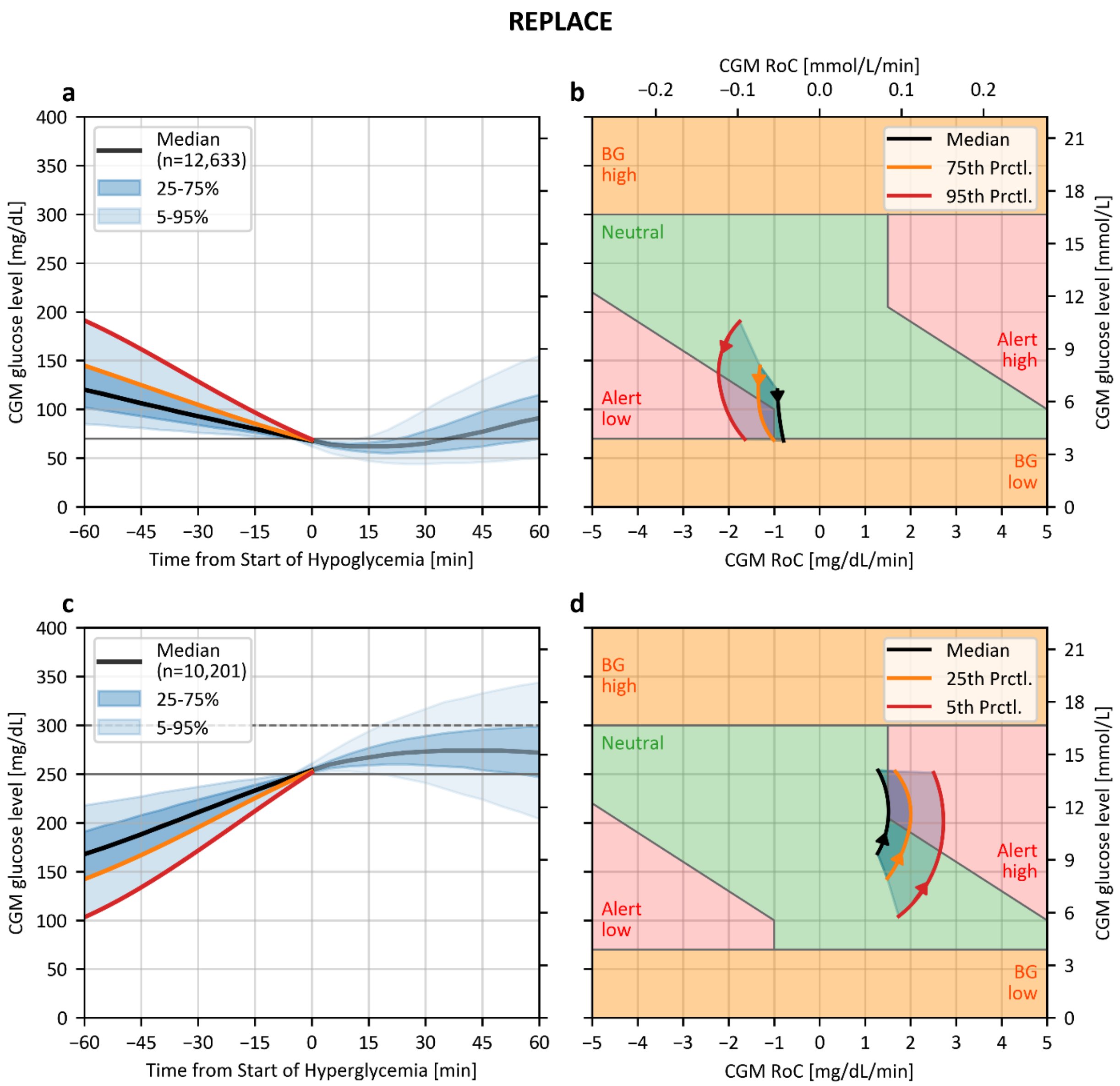

Figure S 1: Results of analyzing the REPLACE-BG dataset, analogous to Figure 3 in the main text.

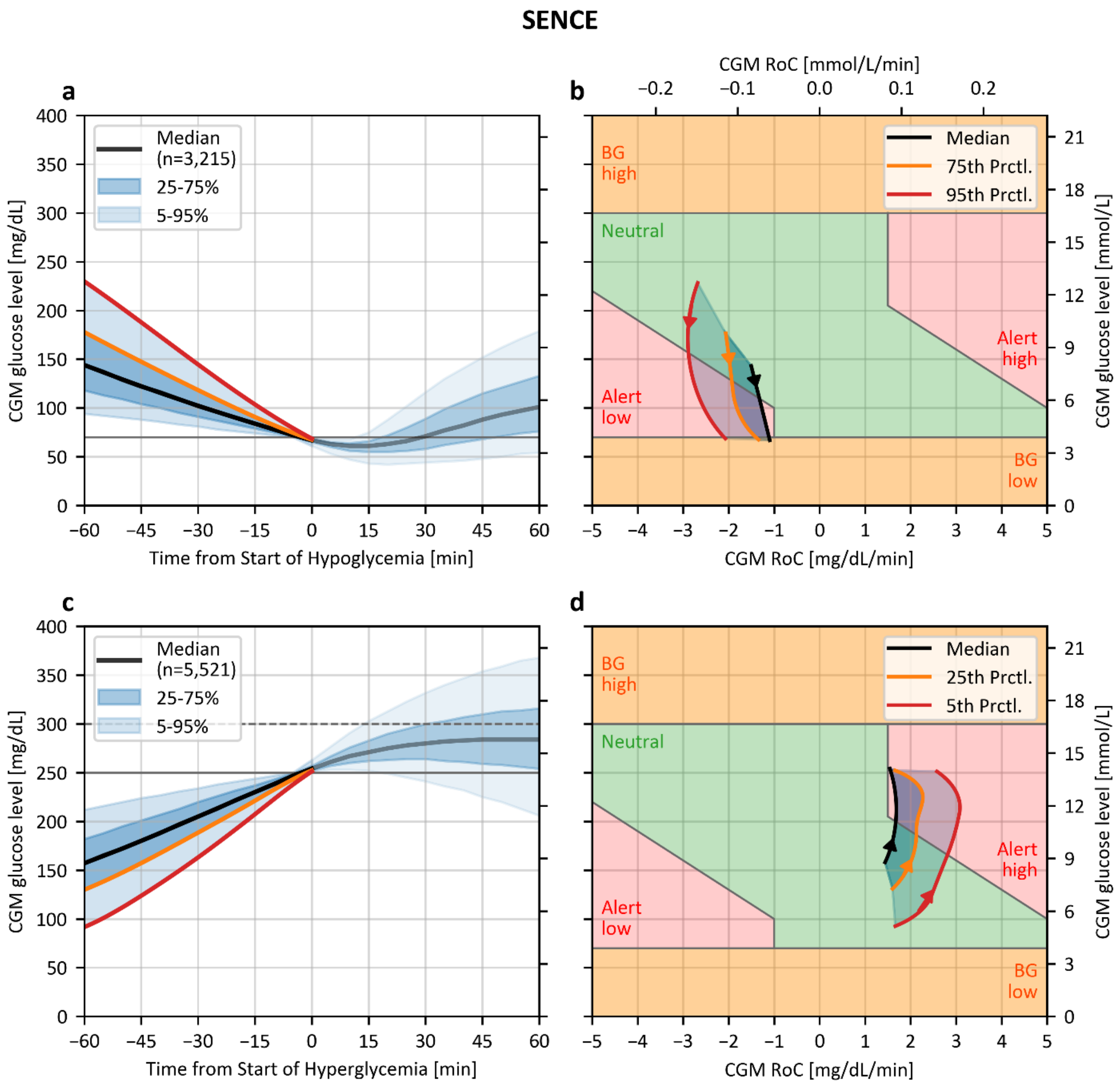

Figure S 2: Results of analyzing the SENCE dataset, analogous to Figure 3 in the main text.

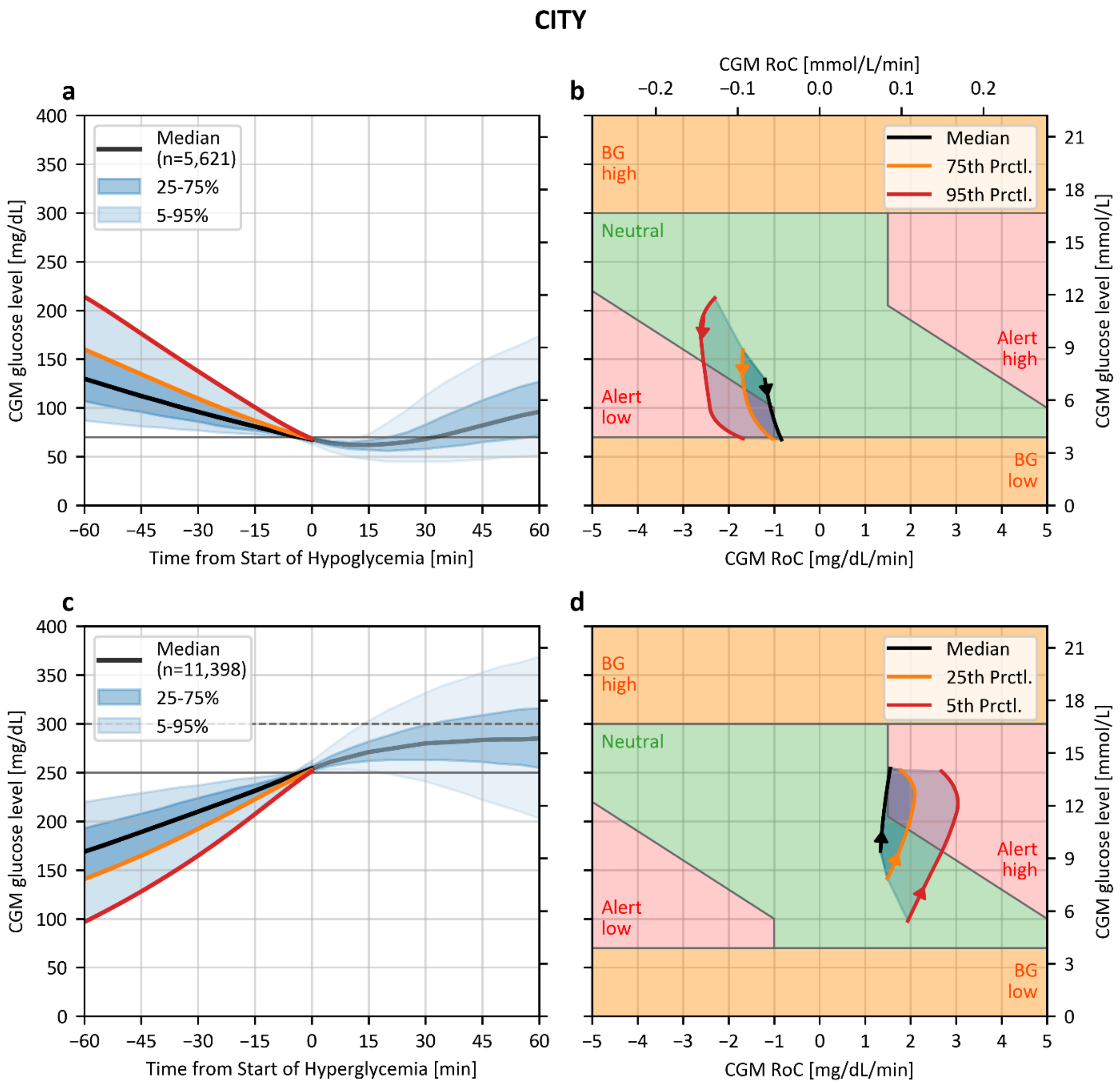

Figure S 3: Results of analyzing the CITY dataset, analogous to Figure 3 in the main text.

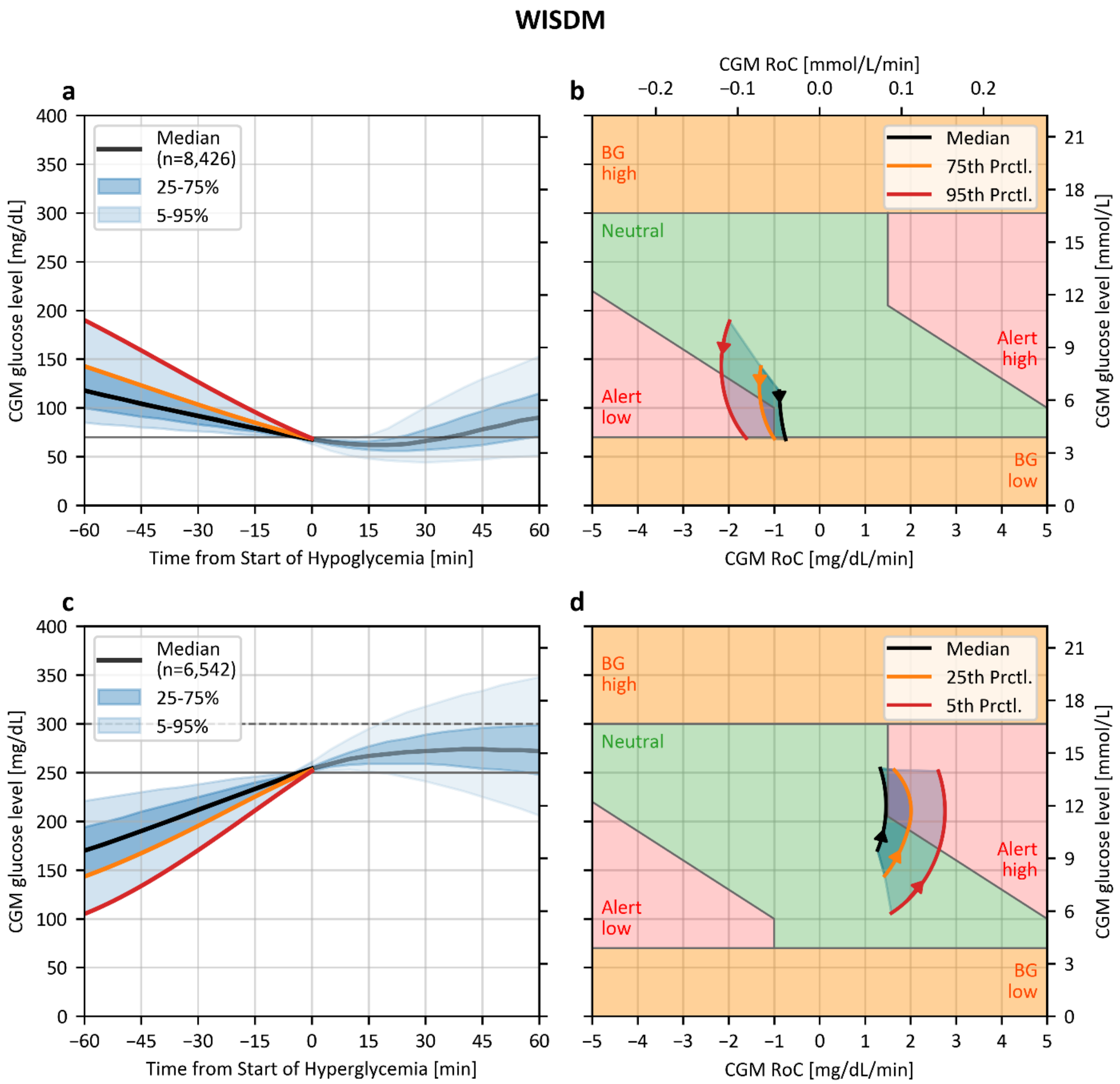

Figure S 4: Results of analyzing the WISDM dataset, analogous to Figure 3 in the main text.

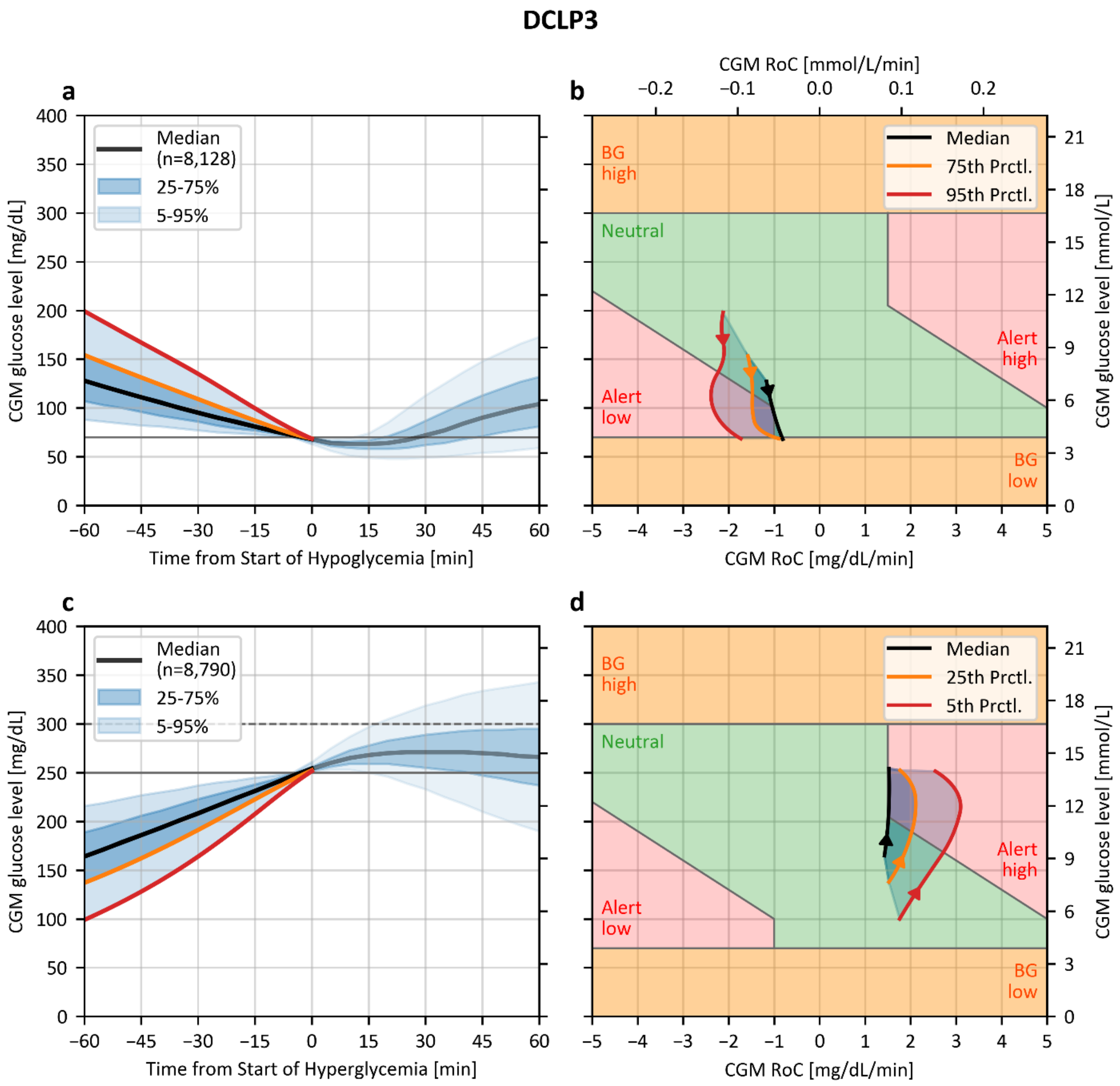

Figure S 5: Results of analyzing the DCLP3 dataset, analogous to Figure 3 in the main text.
